# COVID-19 observations and accompanying dataset of non-pharmaceutical interventions across U.S. universities, March 2020

**DOI:** 10.1371/journal.pone.0240786

**Published:** 2020-10-16

**Authors:** Kevin E. Cevasco, Hayley M. North, Sheryne A. Zeitoun, Rachel N. Wofford, Graham A. Matulis, Abigail F. Gregory, Maha H. Hassan, Aya D. Abdo, David Farris, Amira A. Roess, Michael E. von Fricken

**Affiliations:** 1 Department of Global and Community Health, College of Health and Human Services, George Mason University, Fairfax, Virginia, United States of America; 2 Department of Biology, College of Science, George Mason University, Fairfax, Virginia, United States of America; 3 Environmental Health and Safety Office, George Mason University, Fairfax, Virginia, United States of America; Columbia University, UNITED STATES

## Abstract

**Background:**

The Centers for Disease Control and Prevention (CDC) publishes COVID-19 non-pharmaceutical intervention (NPI) guidance for specific institutional audiences to limit community spread. Audiences include: business, clinical, public health, education, community, and state/local government. The swift, severe, and global nature of COVID-19 offers an opportunity to systematically obtain a national view of how larger institutions of higher education adopted NPI guidance at the onset of the pandemic.

**Method:**

An original database of COVID-19-related university NPI policy changes was compiled. Survey team members manually combed university websites and official statements capturing implementation decisions and dates for five NPI variables from 575 U.S. universities, across 50 states and the District of Columbia, during March of 2020. The universities included in this study were selected from the Department of Education Integrated Postsecondary Education Data System (IPEDS), which provides a set of university explanatory variables. Using IPEDS as the basis for the organizational data allows consistent mapping to event-time and institutional characteristic variables including public health announcements, geospatial, census, and political affiliation.

**Results:**

The dataset enables event-time analysis and offers a variety of variables to support institutional level study and identification of underlying biases like educational attainment. A descriptive analysis of the dataset reveals that there was substantial heterogeneity in the decisions that were made and the timing of these decisions as they temporally related to key state, national, and global emergency announcements. The WHO pandemic declaration coincided with the largest number of university decisions to implement NPIs.

**Conclusion:**

This study provides descriptive observations and produced an original dataset that will be useful for future research focused on drivers and trends of COVID-19 NPIs for U.S. Universities. This preliminary analysis suggests COVID-19 university decisions appeared to be made largely at the university level, leading to major variations in the nature and timing of the responses both between and within states, which requires further study.

## Introduction

When novel pathogens like SARS-CoV-2 spread rapidly, there are limited vaccine and clinical treatments available early in a pandemic. Public health responses to outbreaks of novel pathogens require implementation of nonpharmaceutical interventions (NPI) for individuals and communities to slow the spread of illnesses. The Centers for Disease Control and Prevention (CDC) publishes COVID-19 NPI guidance for specific institutional audiences: business, clinical, public health, education, community, and state/local government [1]. Institutions then interpret and implement NPI guidance, weighed against economic concerns, to determine what extent they will limit where and how people congregate and interact. These interventions typically rely on individual level compliance enacted through institutional policies that limit access to facilities, and utilize persuasive communication.

Understanding how NPIs can contain the pandemic is crucial for balancing public health, economic, and social costs [2]. The CDC pandemic mitigation framework indicates that NPIs are most effective when instituted in an early, targeted, and layered fashion [3, 4]. Research has focused on state and local government implementations as well as individual behaviors, however, limited literature is available focusing on institutional level adoption of NPIs in higher education [5, 6]. Since non-pharmaceutical COVID-19 interventions are, by nature, not randomized, robust data is required to support institutional level study methods [2].

The swift, severe, and global nature of COVID-19 offers a clear opportunity to systematically obtain a national view of higher education NPI adoption. CDC pandemic plans encouraged universities to communicate response measures with staff, students, and key community partners and stakeholders. Therefore, university NPI response policies and specific dates of action were widely available. During March 2020, the Centers for Disease Control and Prevention (CDC), World Health Organization (WHO), Department of Education (ED), state governors, and state and local public health departments all provided guidance on evidence-based mitigation strategies to reduce the incidence and transmission of COVID-19 [7–10]. During the initial stages of the COVID-19 pandemic, universities across the U.S. were compelled to make difficult decisions regarding campus operations and services, often without uniform guidance across state and national leadership. The role schools play in the progression of an outbreak should not be neglected. The Community Preventive Services Task Force recommends pre-emptive, coordinated closure of educational facilities during a severe influenza pandemic (a pandemic with high rates of severe illness such as that experienced in 1918) [11]. This study provides descriptive observations and a baseline dataset containing key decisions related to large university operations and use of NPI interventions at the onset of the COVID-19 pandemic in the United States.

## Methods

An original database of COVID-19-related policy changes was compiled, capturing data from 575 U.S. universities with enrollment of over 5,000 students. Universities offer representation from all 50 states and D.C., a variety of geographic settings, institutional characteristics, and subpopulations. The majority of universities included in this study established COVID-19 webpages to communicate COVID-19 information to students, faculty, contractors, and the local community, allowing data to be combed and extracted by our team. Most NPI decisions were communicated publicly in the form of policy announcements by university leadership timestamped with the date the announcements were made. Publicly available sites captured the chronology of official announcements typically originating from the university president or provost offices. This study examines decisions from February 25th through March 31st, 2020. University social media pages were used as a secondary source for a small proportion of cases, when COVID websites did not contain all relevant data.

The universities included in this study were selected from the Department of Education IPEDS 2018 “First Look Universe” dataset (https://nces.ed.gov/ipeds/datacenter). IPEDS is a system of interrelated surveys conducted annually by the U.S. Department of Education’s National Center for Education Statistics, which gathers information from every college, university, and vocational institution that participates in federal student aid programs. A total of 575 universities in the IPEDS institutional sector were included in this study because they met the following criteria: IPEDS enrollment categories with 5,000 or more students, four-year institution status, and degree granting. These universities represent 7,067,050 students and 2,056,733 faculty/staff members. Four-year institution status was of interest because these universities typically operate study abroad programs, conduct international research, admit a high volume of international students, and have on-campus student housing. Community colleges, online-only institutions, and vocational programs were excluded.

NPI survey variables were based on decisions and recommendations by the Department of Education, CDC, and other national agencies’ published guidance [4, 10–12]. Five outcome variables were selected that affect both students and faculty:

1. “Move online” indicates that the university announced all classes will be conducted in an online/distance learning format, whether for a few weeks or the rest of the semester.
2. “Discourage campus housing” indicates that the university encouraged students to leave on-campus housing.
3. “Cancel travel” indicates that the university decided to cancel/suspend/prohibit all university-sponsored travel.
4. “Close campus” indicates that the university limited campus access to essential/mandatory personnel only.
5. “Remote work” indicates that remote work/telecommuting was the default option for staff/faculty.

The state of each decision (“TRUE” or “FALSE”) and date of the announcement was collected. To be marked as “TRUE”, university leadership was required to have made a clear university-wide policy announcement. Universities with leadership that deferred decisions to the academic unit level were marked as “FALSE”. IPEDS provides supporting data on factors such as faculty and student diversity, public-private governance, religious affiliation, campus setting, geographic location, foreign student presence, academic focus, and on-campus housing. IPEDS and NPI survey data is combined with other data to expand available study variables. COVID-19 state case data were extracted from the ‘COVID Tracking Project’ website (https://covidtracking.com/), which reports current, retrospective, and cumulative numbers by state. Additions to IPEDS data includes Governor and House of Representatives’ party affiliation, university health infrastructure, and U.S. Census divisions. State of emergency dates were sourced from official state records and spring break schedules were extracted from academic calendars.

### Quality control methods

Each NPI survey team member was randomly assigned a list of universities to examine, each of which have unique IPEDS identification numbers and are linked directly to university home webpages. Most universities established COVID-19 webpages and social media presence to communicate COVID-19 information to students, faculty, contractors, and the local community. The primary source for the survey was policy announcements by university leadership labeled with dates the announcements were made. The survey team was provided a guide with various scenarios explaining how to interpret policy announcements to standardize data collection. After the first round of data collection, all universities were reassigned to other team members, with both sets of data compared for concordance. Discrepancies between captured data were then examined by study leads and announcement data were verified to determine final consensus.

### Data management and analysis

Data were entered and managed in Google WorkSheets and Google AppSheet. Descriptive statistics, including range of days between decision dates for individual states and average number of days between decision dates at the state levels were generated using MS Excel and R version 3.6.1 (R Core Team, Vienna, Austria), and Graphs were generated using RStudio version 1.2.5019 (RStudio Inc, Boston, MA).

## Results

**Fig 1:**
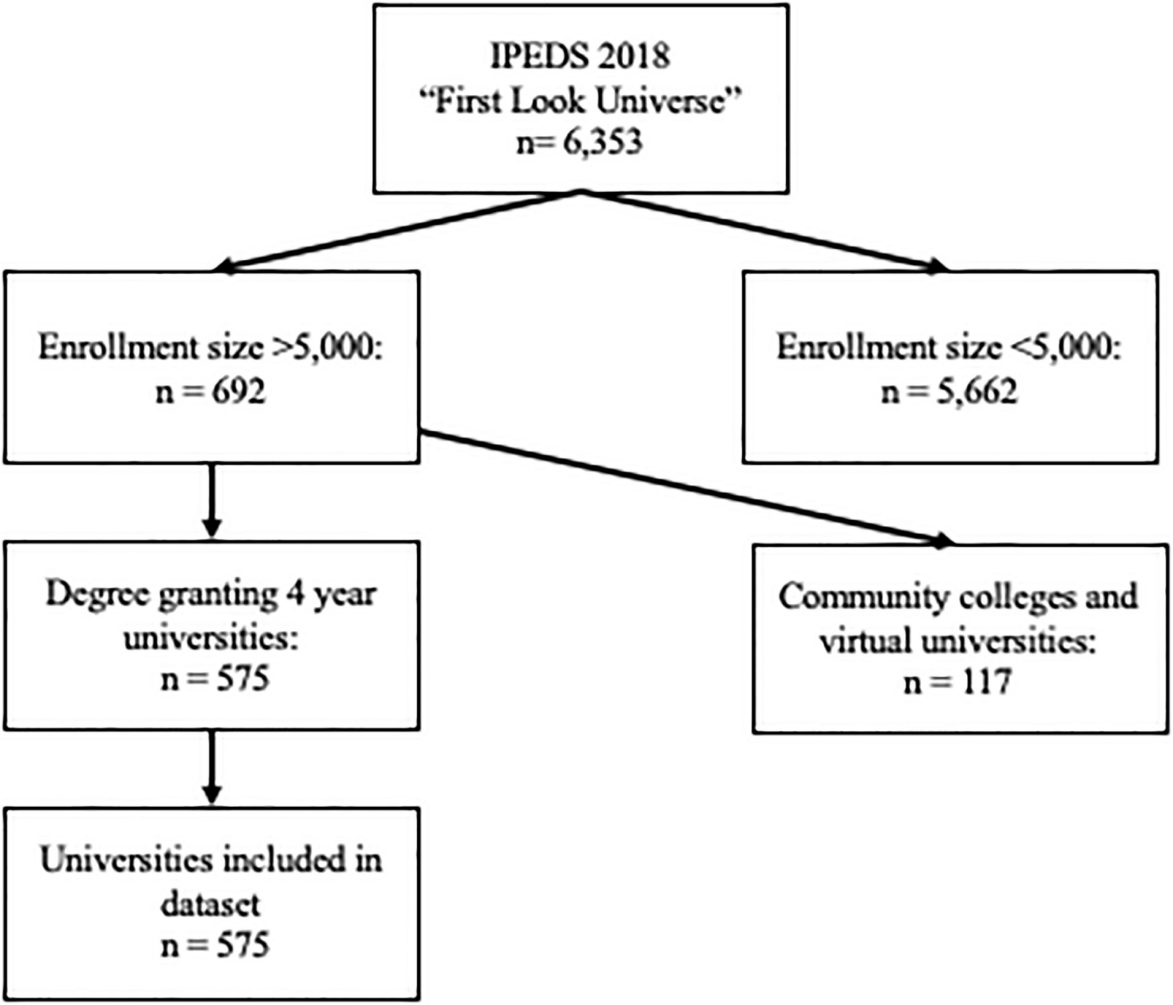
Flow chart depicting university inclusion and exclusion criteria for dataset.

A total of 575 universities were included in this analysis (Fig 1). The NPI survey captured 100% of the university decisions regarding moving online, and between 89% and 95% of the other four NPI variables of interest. Roughly 75% of universities implemented all five NPI mitigation measures, 93% implemented four mitigations, and 98% implemented at least three.

Universities began cancelling international study abroad programs and university-sponsored travel as early as February 25th, with more than 50% canceling international travel by March 11, 2020. All universities that made announcements available concerning their study abroad programs reported cancellation of international travel by March 26. In general, universities quickly announced moving to online learning, with all universities making official announcements between March 4 and March 20. A total of 82% (473/575) of universities announced that they were discouraging on-campus housing between March 9 and March 20th.

Table 1 describes the mean number of positive cases by Census Division when each of the NPI policies were announced. The minimum and maximum provides a range of case prevalence when decisions were announced by universities within each state. Additional information on institutional adoption by state by policy can be found in S1 Table.

**Table 1:** Descriptive statistics mean positive cases and ranges for each Census Division and NPI.

Census Division States (N)	Cancel travel N (%)	Move online N (%)	Campus housing N (%)	Remote work N (%)	Close campus N (%)
	mean (min | max)	mean (min | max)	mean (min | max)	mean (min | max)	mean (min | max)
*New England*	42 (89%)	47 (100%)	44 (94%)	44 (94%)	40 (85%)
CT, ME, MA, NH, RI, VT (47)	0.89	12.53	24.43	282.00	69.95
	(0 | 6)	(0 | 97)	(0 | 156)	(0 | 1060)	(0 | 678)
*Middle Atlantic*	79 (88%)	90 (100%)	78 (87%)	85 (94%)	80 (89%)
NJ, NY, PA (90)	0.88	140.16	1416.56	2635.30	941.80
	(0 | 7102)	(11 | 950)	(11 | 30811)	(6 | 20875)	(6 | 15168)
*East North Central*	75 (94%)	78 (98%)	74 (93%)	76 (95%)	74 (93%)
IN, IL, MI, OH, WI (80)	0.94	129.79	555.92	1654.27	702.68
	(0 | 497)	(3 | 945)	(3 | 9062)	(30 | 10155)	(4 | 9062)
*West North Central*	46 (94%)	49 (100%)	43 (88%)	44 (90%)	41 (84%)
IA, KS, MN, MI, NE, ND, SD (49)	0.94	17.16	38.84	177.88	80.57
	(0 | 128)	(1 | 179)	(1 | 444)	(1 | 1327)	(1 | 502)
*South Atlantic*	86 (87%)	98 (99%)	88 (89%)	91 (92%)	86 (87%)
DE, DC, FL, GA, MD, NC, SC, VA, WV (99)	0.87	34.22	37.78	433.59	169.10
	(0 | 520)	(0 | 186)	(0 | 520)	(7 | 8010)	(0 | 6955)
*East South Central*	37 (93%)	40 (100%)	39 (98%)	34 (85%)	28 (70%)
AL, KY, MS, TN (40)	0.93	14.33	46.33	294.18	122.56
	(0 | 36)	(0 | 228)	(0 | 587)	(10 | 1834)	(8 | 957)
*West South Central*	58 (92%)	62 (98%)	51 (81%)	59 (94%)	54 (86%)
AR, LA, OK, TX (63)	0.92	26.11	158.57	472.17	187.51
	(0 | 143)	(3 | 194)	(6 | 1172)	(22 | 2877)	(10 | 1731)
*Mountain*	36 (95%)	38 (100%)	29 (76%)	36 (95%)	31 (82%)
AZ, CO, ID, NM, MN, UT, NV, WY (38)	0.95	18.24	53.66	182.71	52.89
	(0 | 245)	(0 | 160)	(0 | 216)	(11 | 912)	(0 | 216)
*Pacific*	60 (87%)	68 (99%)	56 (81%)	65 (94%)	64 (93%)
AK, CA, HI, OR, WA (69)	0.87	362.04	838.18	1183.16	653.26
	(0 | 4551)	(0 | 2218)	(0 | 4146)	(12 | 5923)	(1 | 3810)
Grand Total	519 (90%)	570 (99%)	502 (87%)	534 (93%)	498 (87%)
(575)	205	97	430	403	1012
	(0 | 7102)	(0 | 2218)	(0 | 30811)	(0 | 15168)	(1 | 20875)

**Fig 2:**
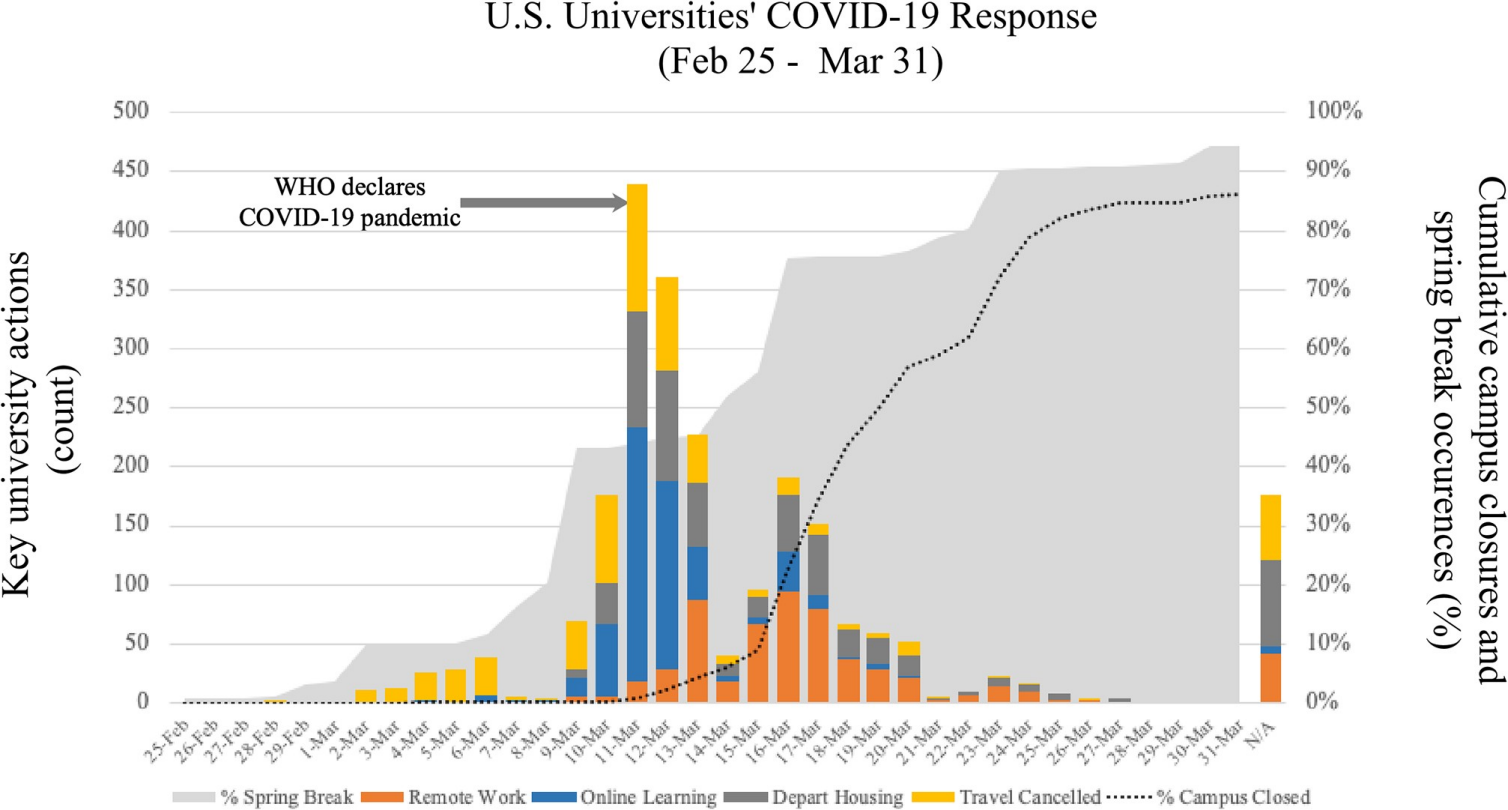
Timing and count of university NPI decisions against backdrop of spring break and campus closure.

The histogram in Fig 2 depicts the five university NPI decisions of interest over time. The WHO pandemic declaration coincides with the most active announcement day, with 73% of all decisions to move learning to remote delivery methods made between the WHO declaration on March 11^th^ and the U.S. national emergency declaration on March 13^th^. The CDC Interim Pre-pandemic Planning Guidance suggests implementing NPIs when WHO declares a Pandemic Period [4]. The four other variables of interest show a broad range of implementation dates for institutional NPIs. Decisions that mostly affected students, including canceling travel, shifting to remote learning, and limiting on-campus housing, occurred before decisions that mostly affected faculty/staff, which included implementing remote work and closing campus.

Supplementary forest plots visualize the timing of decisions by state in relation to the date that each state declared a state of emergency (SOE), the WHO declared a pandemic, and U.S. declared a national emergency (S1–S5 Figs). Forest plots are arranged in a cascading state SOE order, where early states are displayed at the top of the list and late state SOEs are displayed near the bottom. The wide bars in the forest plots show a great variation in the timing of both decisions both within and between states, where only 17% (86/502) of universities discouraged on-campus housing prior to state SOE declaration. Only 22.9% (137/502) of universities that eventually discouraged on-campus housing waited until at least a week after the governor declared an SOE.

## Discussion

There are many factors that may explain the wide variation in decision-making observed in this study. A systematic review of school closure and management practices found a dearth of policy-relevant data on the implementation of school social distancing during coronavirus outbreaks [13]. At the higher education level, a study of university COVID-19 responses in 20 countries shows responses have been diverse [6]. This novel study provides a comparatively rich set of NPI variables, and observations that allows for ongoing analysis. The data collected supports a variety of study methods (case-control, survival analysis, and difference in differences) with extensive institutional and time/event variables [2, 14]. The variation in timing and alignment with public health declarations show the value in a national dataset. Pandemic models require local differentiation and adaptation once released, and understanding how NPIs are interpreted by institutions that implement them needs to be further studied [15]. Roughly 73% of universities moved to on-line learning over a three-day period, which may indicate institutional isomorphism. This study includes a dataset with four additional NPI decisions where the results show a variety of university behavior. Future studies can use this dataset to address confounding and interaction from factors such as spring break, public health declarations, and university-linked explanatory variables.

Government policy and political partisanship can have significant influence on universities’ implementation of pandemic response actions [16]. Clear and concise leadership is needed at the state and federal levels. A 2006 study found that influenza pandemic response plans and policies often lacked specific “triggers” to implement outbreak response measures related to students and faculty [7]. On March 9^th^, the CDC released Guidance for Institutions of Higher Education with Students Participating in International Travel or Study Abroad Program followed by the March 11^th^ WHO pandemic declaration. Additionally, on March 13^th^, the White House declared a national emergency. This week was the most active in terms of university response, and methods must therefore address a variety of event variables.

### Limitations

All data extracted from official announcements captures information that was publicly shared, which may have resulted in an underrepresentation of decisions communicated by email only. Nor does this review capture internal decision timelines for universities, as it only captures public announcements regarding decisions. Some NPI survey data was subject to interpretation of university actions. The survey team was provided a guide with various scenarios explaining how to interpret policy announcements in order to standardize data collection, with quality control checks conducted by study leads to reach a consensus. When specific dates or other information were not included on announcement pages, efforts were made to confirm timing by searching social media and other public sources. The data is based on final decision as of 3/31/20. In general, once major decisions were made, we saw no evidence of backtracking and reduction of restrictions for the month of March. Furthermore, only universities form the IPEDS categories of 5,000 or more enrolled students were included. Therefore, the response and behavior of smaller universities, and those in small town and rural locations may differ.

## Conclusions

Local versus national NPI implementation decisions are beneficial in the sense that universities could implement NPIs based on community spread or exposure from student and faculty who had recently travel to endemic areas. However, uncoordinated guidance and declarations from different public health authorities may complicate the decision process in the presence of inconsistent guidance from government leadership. Policy decisions were often made during the on-schedule or extended spring breaks for universities, when a large number of students may have been traveling. In this sense, robust data is required to understand if the timing of NPI decisions may have avoided the movement of millions of students back onto campus and ensuing instances of community spread. The dataset provided may prove useful in examining parallels between universities that were early actors or laggards in implementing NPIs this spring and if that correlates to risk profiles regarding future university decisions related to operations, safety, response, and shutdowns due to cluster outbreaks on campus.

## Supporting information

S1 Fig University cancel travel announcement dates with public health declarations. (TIF)

S2 Fig University move on-line announcement dates with public health declarations. (TIF)

S3 Fig University campus housing announcement dates with public health declarations. (TIF)

S4 Fig University remote work announcement dates with public health declarations. (TIF)

S5 Fig University close campus effective dates with public health declarations. (TIF)

S1 Table Descriptive statistics mean positive cases and ranges for each state and NPI. (DOCX)

S1 Appendix Survey instrument. (DOCX)

S1 File Data dictionary. (DOCX)

S1 Dataset (XLSX)
