# Supplementary figures and images for: COVID-19 observations and accompanying dataset of non-pharmaceutical interventions across U.S. universities, March 2020
Source: PLoS One. 2020 Oct 16;15(10):e0240786. doi: 10.1371/journal.pone.0240786 (PMC7567344; doi:10.1371/journal.pone.0240786)

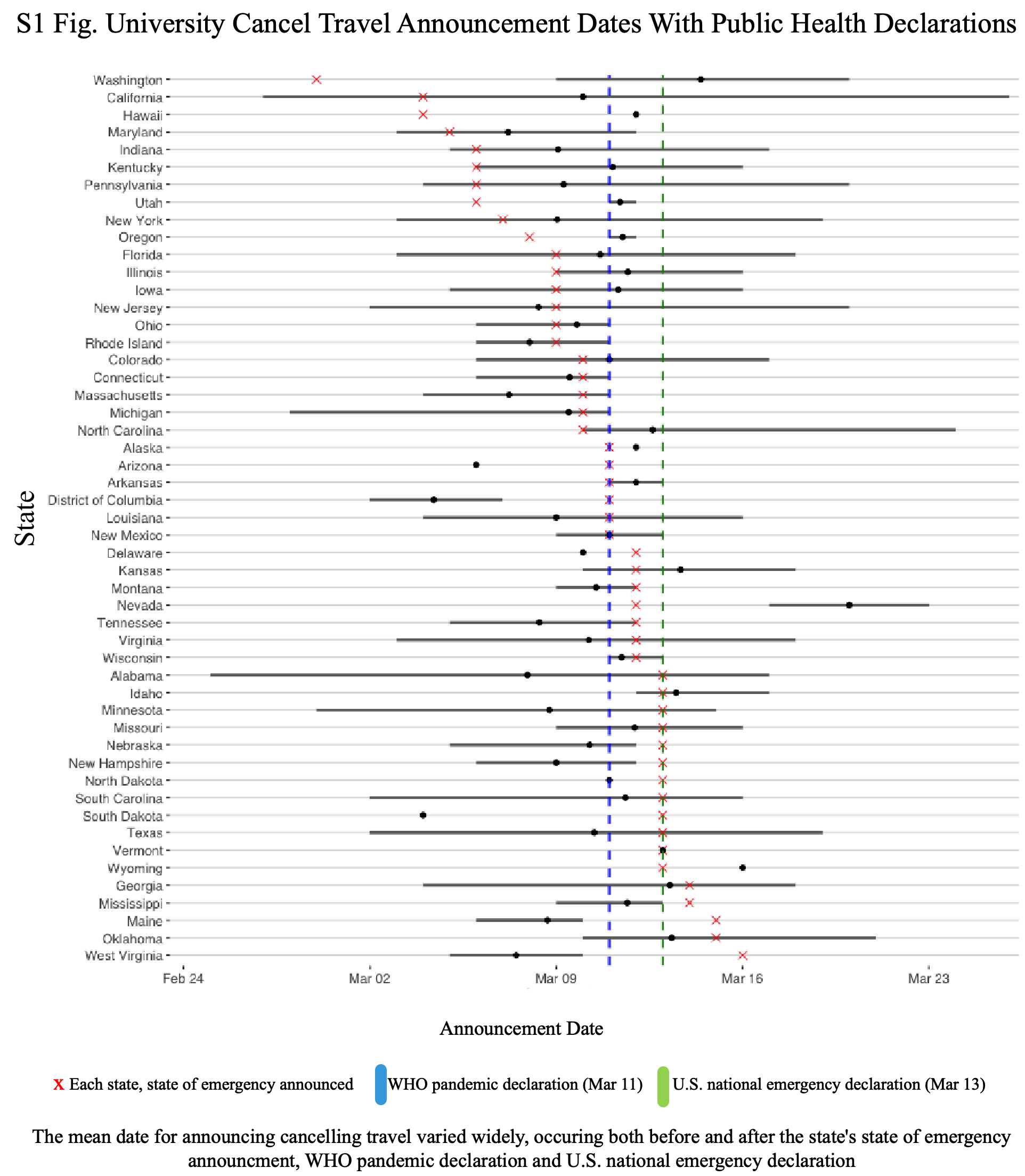

Supplement: S1 Fig — (TIF) [file pone.0240786.s001.tif]

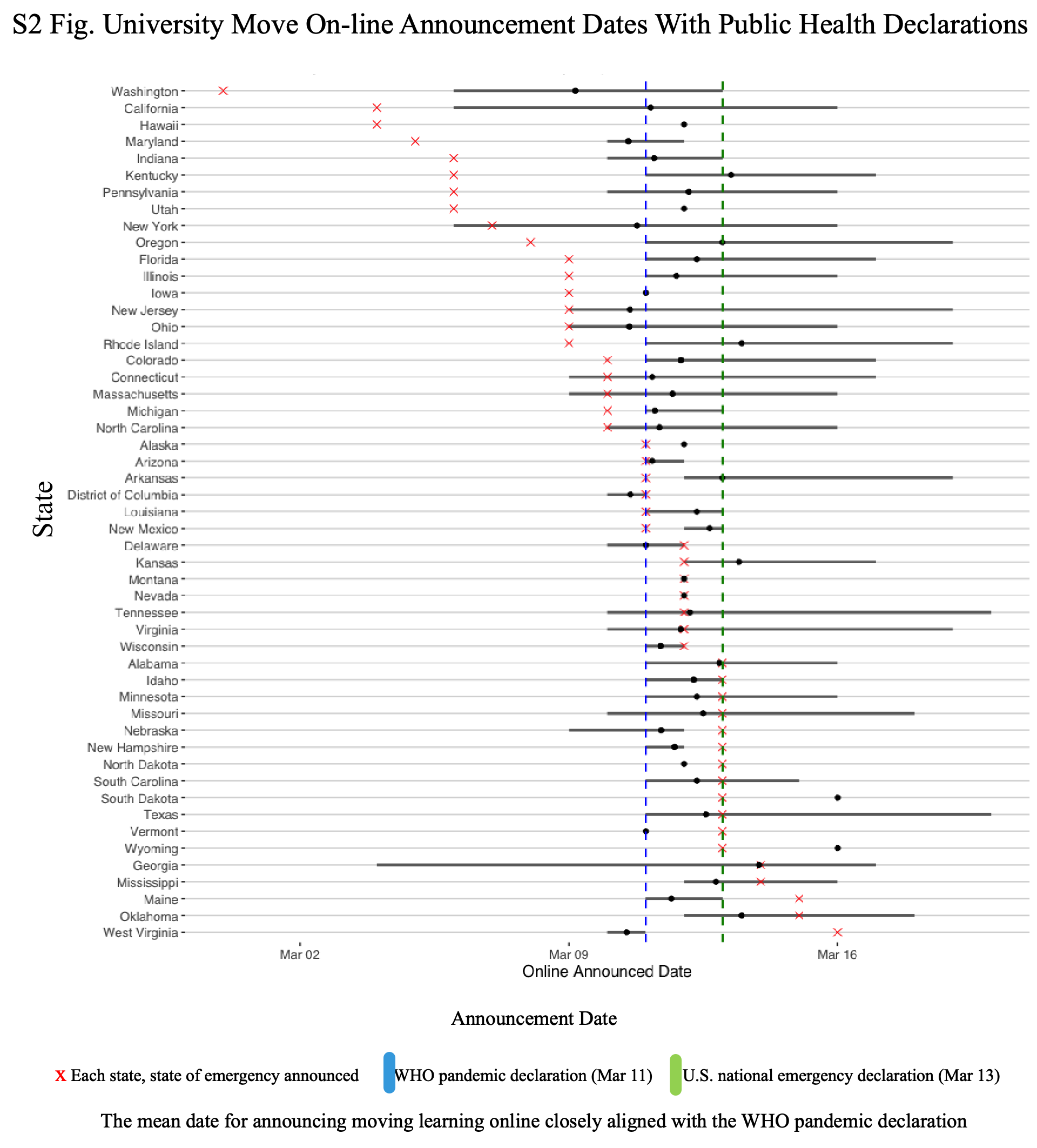

Supplement: S2 Fig — (TIF) [file pone.0240786.s002.tif]

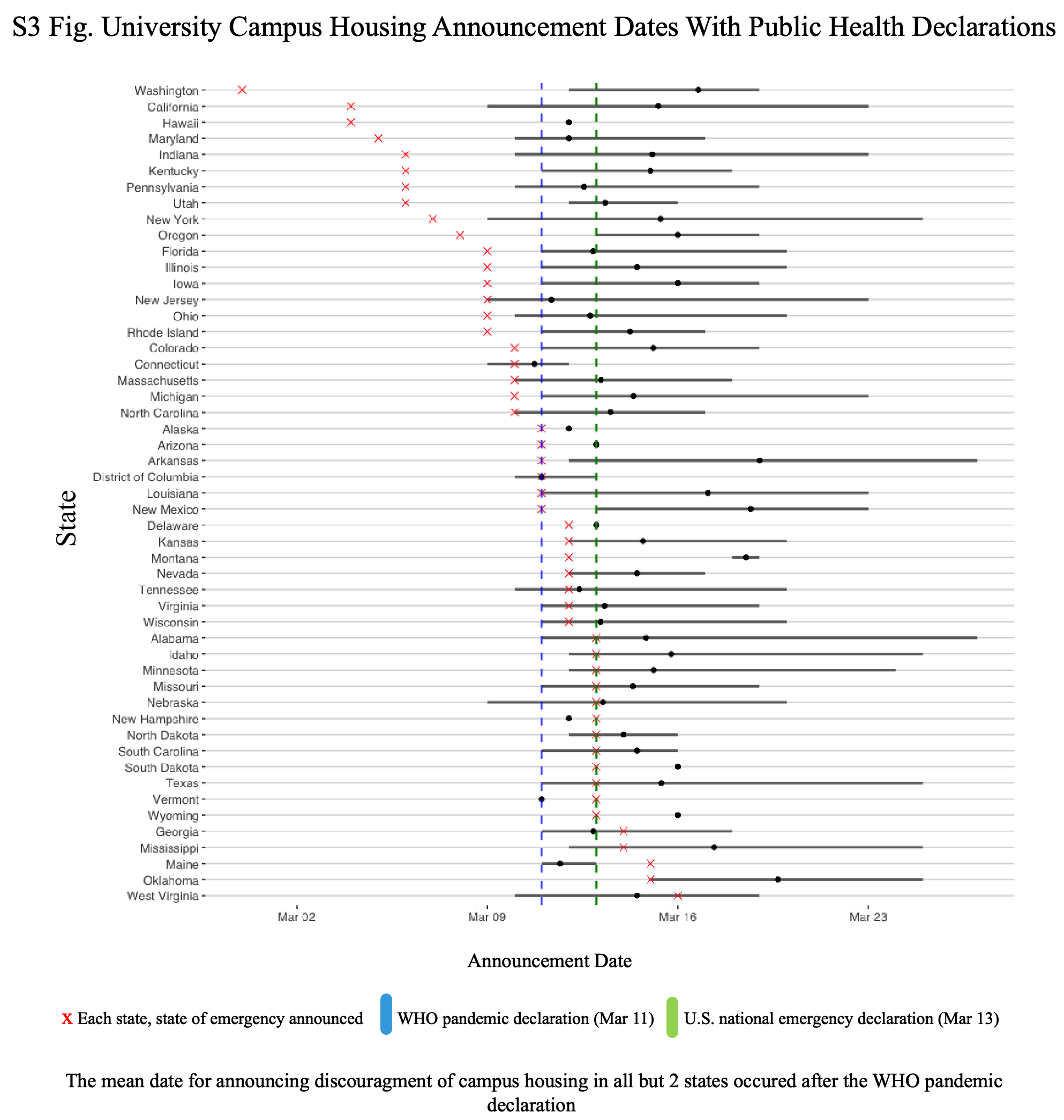

Supplement: S3 Fig — (TIF) [file pone.0240786.s003.tif]

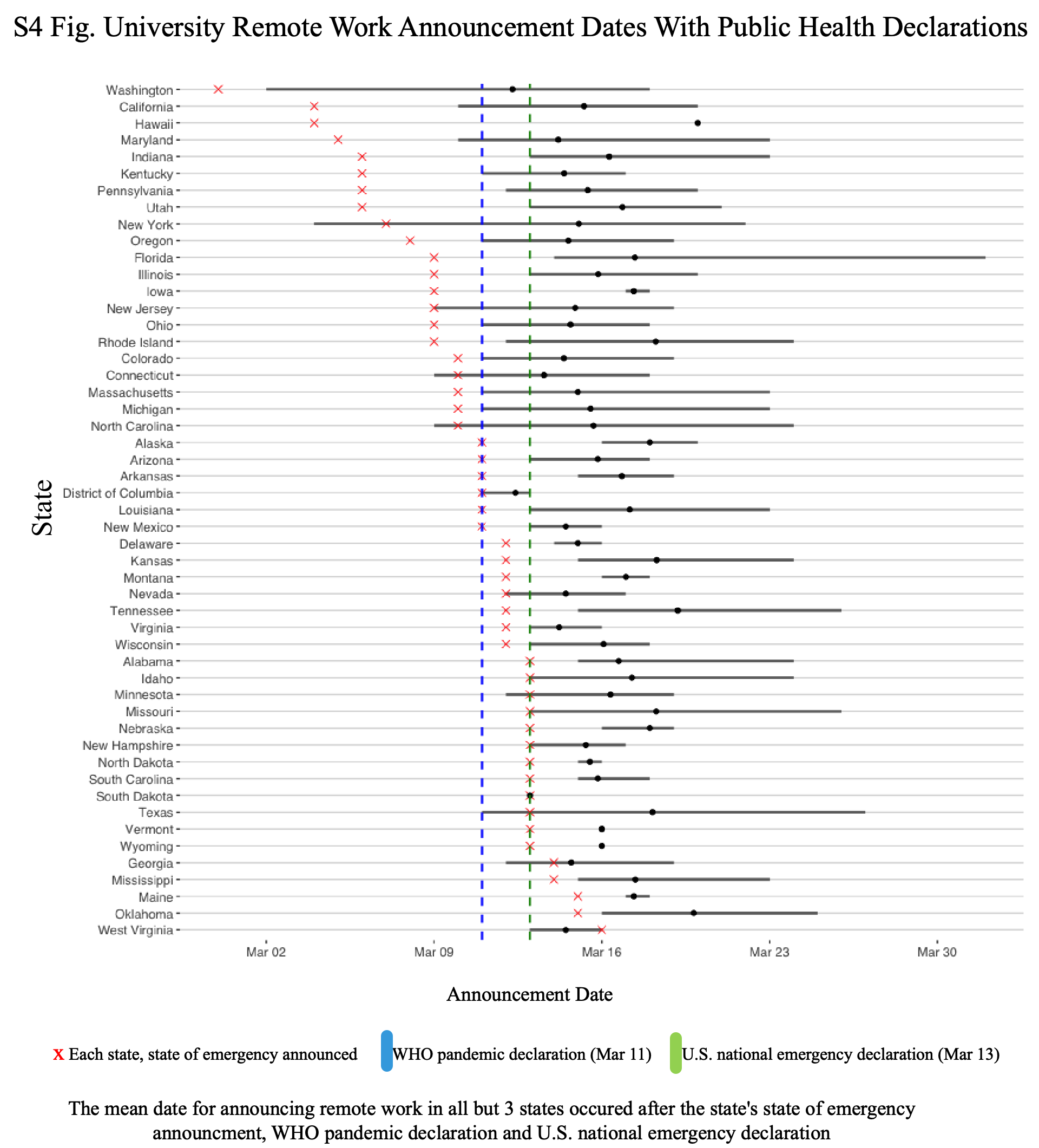

Supplement: S4 Fig — (TIF) [file pone.0240786.s004.tif]

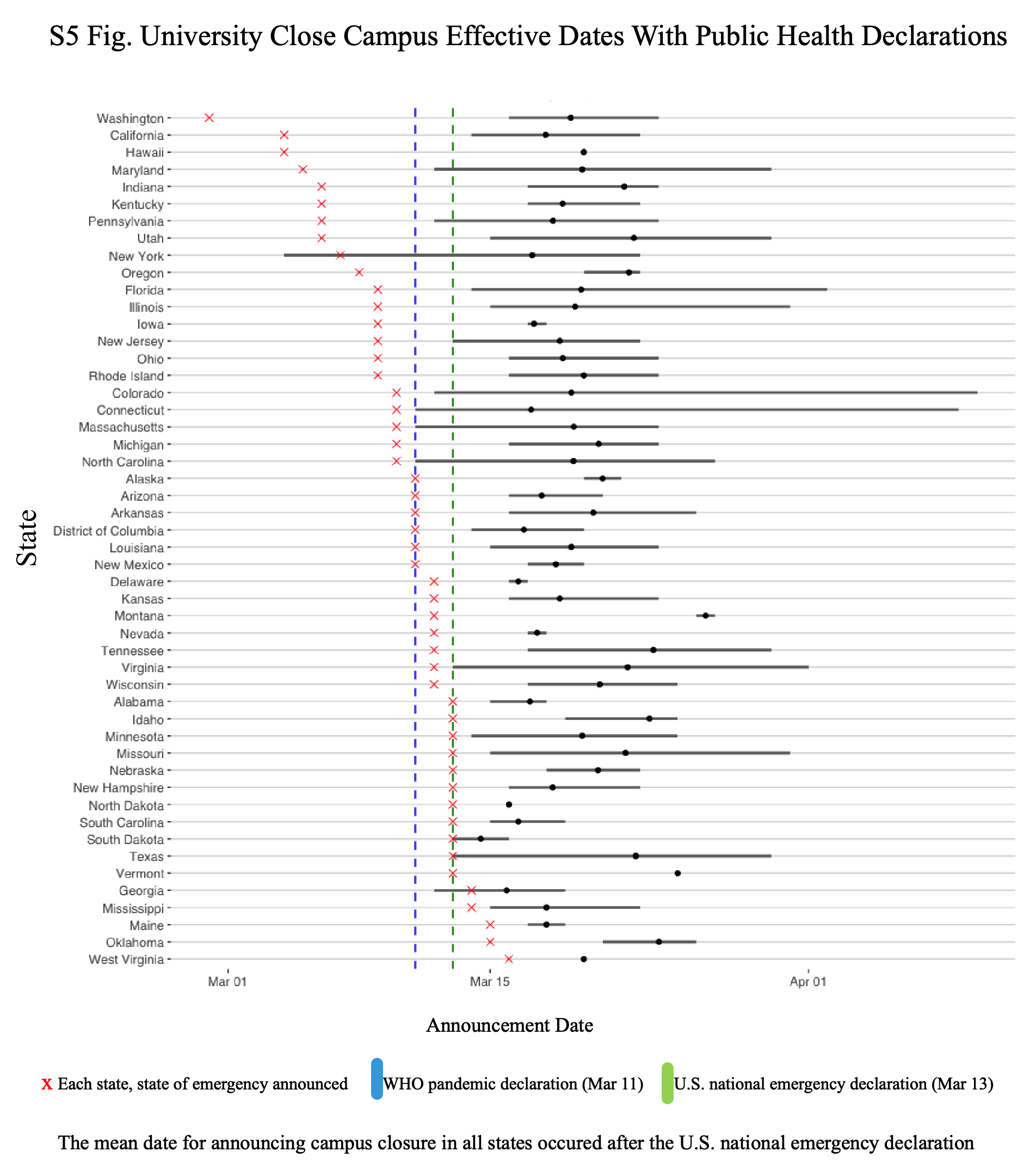

Supplement: S5 Fig — (TIF) [file pone.0240786.s005.tif]
