# Supplementary material for: COVID-19 observations and accompanying dataset of non-pharmaceutical interventions across U.S. universities, March 2020
Source: PLoS One. 2020 Oct 16;15(10):e0240786. doi: 10.1371/journal.pone.0240786 (PMC7567344; doi:10.1371/journal.pone.0240786)
Supplement: S1 Table — (DOCX) [file pone.0240786.s006.docx]

S1 Table: Descriptive statistics mean positive cases and ranges for each state and NPI

| State (N) | Cancel Travel  Mean (Min\|Max) | Move Online Mean (Min\|Max) | Discourage Campus Housing  Mean (Min\|Max) | Remote Work  Mean (Min\|Max) | Close Campus  Mean (Min\|Max) |
| --- | --- | --- | --- | --- | --- |
| Alabama (12) | 3 (0\|36) | 5 (0\|28) | 61 (0\|587) | 43 (12\|215) | 37 (12\|46) |
| Alaska (2) | 0 (0\|0) | 0 (0\|0) | 0 (0\|0) | 7 (1\|12) | 17 (12\|22) |
| Arizona (6) | 8 (3\|9) | 9 (9\|9) | 9 (9\|9) | 18 (9\|28) | 42 (18\|104) |
| Arkansas (7) | 5 (0\|9) | 12 (6\|46) | 154 (6\|381) | 27 (16\|46) | 134 (22\|335) |
| California (47) | 213 (0\|3006) | 169 (60\|335) | 454 (114\|1733) | 374 (133\|1063) | 699 (252\|1733) |
| Colorado (12) | 33 (2\|160) | 47 (28\|160) | 118 (28\|216) | 103 (28\|216) | 255 (45\|912) |
| Connecticut (12) | 2 (0\|3) | 6 (1\|41) | 3 (1\|6) | 19 (1\|68) | 38 (3\|194) |
| Delaware (2) | 2 (0\|4) | 2 (0\|4) | 4 (4\|4) | 7 (6\|8) | 12 (8\|16) |
| District of Columbia (5) | 0 (0\|0) | 5 (5\|5) | 6 (5\|10) | 9 (5\|10) | 29 (10\|71) |
| Florida (24) | 71 (0\|520) | 52 (28\|186) | 78 (28\|520) | 544 (77\|6955) | 1215 (77\|8010) |
| Georgia (20) | 74 (2\|197) | 79 (2\|146) | 49 (22\|197) | 95 (31\|287) | 132 (31\|287) |
| Hawaii (1) | 2 (2\|2) | 2 (2\|2) | 2 (2\|2) | 26 (26\|26) | 26 (26\|26) |
| Idaho (4) | 2 (0\|7) | 0 (0\|0) | 18 (0\|73) | 16 (0\|50) | 58 (11\|73) |
| Illinois (20) | 26 (7\|93) | 26 (19\|93) | 155 (19\|585) | 174 (32\|585) | 744 (64\|5994) |
| Indiana (15) | 7 (0\|30) | 10 (6\|12) | 37 (6\|259) | 57 (12\|259) | 238 (30\|365) |
| Iowa (3) | 13 (0\|22) | 13 (13\|13) | 27 (13\|38) | 25 (23\|29) | 25 (23\|29) |
| Kansas (7) | 7 (1\|16) | 7 (4\|15) | 13 (4\|44) | 31 (8\|98) | 32 (11\|98) |
| Kentucky (9) | 9 (0\|21) | 13 (8\|22) | 17 (8\|26) | 15 (8\|22) | 40 (22\|104) |
| Louisiana (12) | 34 (0\|137) | 24 (6\|36) | 325 (6\|1172) | 258 (36\|1172) | 541 (90\|1388) |
| Maine (3) | 0 (0\|0) | 1 (0\|3) | 1 (0\|3) | 36 (32\|43) | 43 (32\|53) |
| Maryland (11) | 4 (0\|12) | 8 (6\|12) | 18 (6\|57) | 47 (6\|288) | 283 (12\|1413) |
| Massachusetts (23) | 0 (0\|0) | 17 (0\|97) | 41 (0\|156) | 113 (0\|678) | 472 (0\|1060) |
| Michigan (17) | 408 (0\|497) | 547 (497\|945) | 2325 (497\|9062) | 2815 (497\|9062) | 6999 (1933\|10155) |
| Minnesota (12) | 22 (0\|128) | 54 (21\|179) | 147 (43\|444) | 202 (43\|287) | 327 (90\|502) |
| Mississippi (6) | 2 (0\|4) | 3 (1\|12) | 87 (1\|377) | 62 (10\|249) | 90 (10\|249) |
| Missouri (18) | 2 (0\|6) | 2 (1\|13) | 7 (1\|24) | 61 (2\|502) | 248 (5\|1327) |
| Montana (2) | 1 (0\|1) | 1 (1\|1) | 11 (10\|12) | 9 (7\|10) | 90 (71\|108) |
| Nebraska (5) | 8 (0\|10) | 9 (3\|10) | 14 (3\|32) | 24 (18\|27) | 38 (24\|50) |
| Nevada (2) | 150 (55\|245) | 7 (7\|7) | 31 (7\|55) | 31 (7\|55) | 55 (55\|55) |
| New Hampshire (4) | 4 (2\|6) | 6 (4\|6) | 6 (6\|6) | 12 (6\|17) | 35 (13\|78) |
| New Jersey (17) | 75 (0\|890) | 59 (11\|742) | 190 (11\|2844) | 226 (11\|742) | 870 (50\|2844) |
| New Mexico (3) | 4 (0\|10) | 8 (5\|10) | 39 (10\|65) | 14 (10\|17) | 33 (23\|43) |
| New York (48) | 1597 (0\|7102) | 227 (33\|950) | 2953 (142\|30811) | 1616 (6\|15168) | 4218 (6\|20875) |
| North Carolina (18) | 36 (7\|398) | 9 (7\|33) | 21 (7\|40) | 68 (2\|398) | 230 (7\|763) |
| North Dakota (2) | 0 (0\|0) | 1 (1\|1) | 1 (1\|1) | 1 (1\|1) | 1 (1\|1) |
| Ohio (15) | 3 (0\|4) | 7 (3\|50) | 28 (3\|169) | 37 (4\|88) | 200 (50\|564) |
| Oklahoma (6) | 11 (2\|53) | 7 (3\|29) | 56 (9\|164) | 57 (10\|164) | 143 (53\|248) |
| Oregon (7) | 19 (19\|19) | 32 (19\|88) | 59 (30\|88) | 49 (19\|88) | 176 (114\|191) |
| Pennsylvania (25) | 22 (0\|268) | 29 (12\|76) | 37 (12\|185) | 85 (22\|268) | 230 (22\|851) |
| Rhode Island (4) | 4 (3\|5) | 23 (5\|61) | 21 (5\|33) | 82 (13\|148) | 85 (22\|148) |
| South Carolina (6) | 14 (0\|28) | 11 (9\|19) | 19 (9\|28) | 29 (19\|60) | 36 (19\|60) |
| South Dakota (2) | 5 (0\|10) | 10 (10\|10) | 10 (10\|10) | 9 (9\|9) | 10 (9\|10) |
| Tennessee (13) | 4 (1\|18) | 30 (7\|228) | 32 (7\|228) | 277 (39\|957) | 740 (73\|1834) |
| Texas (38) | 25 (0\|143) | 33 (21\|194) | 125 (21\|974) | 213 (21\|1731) | 578 (39\|2877) |
| Utah (8) | 3 (0\|4) | 4 (4\|4) | 16 (4\|39) | 56 (6\|136) | 350 (28\|806) |
| Vermont (1) | 5 (5\|5) | 2 (2\|2) | 2 (2\|2) | 17 (17\|17) | 160 (160\|160) |
| Virginia (11) | 15 (0\|77) | 19 (8\|94) | 31 (9\|94) | 38 (30\|51) | 410 (30\|1484) |
| Washington (12) | 2532 (731\|4551) | 1372 (813\|2218) | 3412 (1921\|4146) | 2272 (450\|3810) | 4261 (3107\|5923) |
| West Virginia (2) | 0 (0\|0) | 0 (0\|0) | 1 (0\|2) | 0 (0\|0) | 7 (7\|7) |
| Wisconsin (13) | 6 (3\|23) | 5 (3\|9) | 40 (3\|235) | 65 (23\|121) | 323 (83\|667) |
| Wyoming (1) | 3 (3\|3) | 3 (3\|3) | 3 (3\|3) | 3 (3\|3) | 0 (0\|0) |
| **Total (575)** | 205 (0\|7102) | 97 (0\|2218) | 430 (0\|30811) | 403 (0\|15168) | 1012 (1\|20875) |
