# Supplementary material for: COVID-19 observations and accompanying dataset of non-pharmaceutical interventions across U.S. universities, March 2020
Source: PLoS One. 2020 Oct 16;15(10):e0240786. doi: 10.1371/journal.pone.0240786 (PMC7567344; doi:10.1371/journal.pone.0240786)
Supplement: S1 Appendix — (DOCX) [file pone.0240786.s007.docx]

**S1 Appendix – Survey Instrument**

# Travel Cancellation

*Variable: Study_abroad_canceled?*University cancellation of travel is defined as a suspension of all university sponsored traveled to all countries outside the United States of America. Specific country travel bans (i.e. CDC level countries) were not included as a university cancellation of travel because country specific bans frequently aligned with Department of State announcements and were not considered decisive action by university leadership.
Values: “True” if announcement was made, “False” if evidence that travel was still permitted at the end of the survey period, “Blank” if no evidence found.

- *Variable: Abroad_cancel_announced_date* The date the university published an announcement about suspending all university sponsored travel.
  Values: Date or blank
- *Variable: Abroad_cancel_effective_date*
  The date effective was the date specified by the university that would begin the suspension of all university sponsored travel. If “effective immediate” was specified, the date of the announcement was used. If an effective date was not specified, the date of the announcement was used.
  Values: Date or blank

# Move to Online Learning

*Variable: Move_Online?*

If the university announced all courses would be provided in an online or distance learning format, regardless of the length of the transition, it was considered a move to online learning. If a university announced that all classes except those approved (i.e. labs, studios) are moved to an online format, it was still considered a university decision to move online.

Values: “True” if announcement was made, “False” if evidence on campus learning was still permitted at the end of the survey period, “Blank” if no evidence found.

- *Variable: Online_announced_date*The date the initial communication was made that the university would be moving to an online learning format was published. If a decision to move online temporarily occurred before the decision to move online for the rest of the semester/quarter, the announcement of the initial transition was recorded.
  Values: Date or blank
- *Variable: Online_effective_date* A specified effective date, or date of the communication if stated “effective immediately”, was used as the online effective date. If the announcement was made during a scheduled university break or weekend and an effective date was not communicated, the next date courses resumed was used as the effective date.
  Values: Date or blank
- Variable: Class_suspended?
  University suspension of classes is defined as the cancellation of scheduled courses, to include an extension or a university break. Additionally, if previously online courses were given permission to continue but on-campus/in-person courses were cancelled, it was also considered a suspension of classes. If the decision to cancel classes was deferred to the faculty member it was not considered a suspension of classes. Neither was it considered a suspension of classes if the weekend or scheduled school break was used as a transitional period between in-person and online learning.
  Values: “True” if announcement was made, “False” if evidence that no suspension was in place by the end of the survey period, “Blank” if no evidence found.
- *Variable: Full_semester_online?*A communication or decision from the university that courses would be delivered in a remote or online format for the remainder of the spring 2020 semester or quarter. If interim decisions were made on an ongoing basis throughout the semester, it was not considered a decision to move online for the remainder of the semester/quarter.
  Values: “True” if announcement was made, “False” if evidence that no announcement of full semeseter online was made by the end of the survey period, “Blank” if no evidence found.

# University Discourages On-Campus Housing

*Variable: Dorms_discouraged?*

University communication that states students should not return to on-campus housing, should leave on-campus housing, or return to their permanent residence. Offers of financial refunds for housing costs were included as an effector to discourage students being in on-campus housing.

Values: “True” if announcement was made, “False” if evidence that no discouragement of on-campus housing was made by the end of the survey period, “Blank” if no evidence found.

- *Variable: dorms_announced_date* The date provided on the university announcement that communicated the university discourages on-campus housing was used as the announcement date. If a university communication did not meet the above requirements or was not provided, no date was input as an announcement date.
  Values: Date or blank

# Campus Closed

*Variable: Essential_personnel?*

Announcement that campus was closed except for essential personnel, mandatory personnel, or employees whose job cannot be done remotely. Any university communication that stated only essential personnel should report to campus, or that only essential personnel have permission to be on-campus grounds were marked as campus access limited to essential personnel. A university communication that indicated a transition to a tiered system of operation was also included as essential personnel only.

Values: “True” if announcement was made, “False” if evidence that no campus closure was in place by the end of the survey period, “Blank” if no evidence found.

- *Effective Date.*
  *Variable: Campus_essential_date*
  The date that specified essential or mandatory personnel only were allowed on campus. If “effective immediate” was used in the university communication, the date the communication was published was used. When a specific start date was provided, regardless of the communication publish date, the specified date was used. If no date was specified and “effective immediate” was not present in the communication, the date of the communication was used.
  Values: Date or blank

# Remote Work

*Variable: Voluntary_telecommute?*
Remote working is defined as teleworking, remote working, or working from home. A campus communication from the university that stated faculty and staff are encouraged, welcome or allowed to work remotely was used. If the university required faculty or staff to use paid time off, or speak with their supervisors or get approval from their manger, this was not included as decisive encouragement from the university. No response was recorded if the university transitioned to essential personnel only before an announcement was made about remote work.

Values: “True” if announcement was made, “False” if evidence that no remote work declaration was made by the end of the survey period, “Blank” if no evidence found.

- *Policy Announcement Date*.
  *Variable: Campus_Telecommute_Date*
  The date a communication was published that met the above criteria was used as the date encouraged. The date of a communication that stated remote work was a possibility or to prepare to move to remote work was not recorded as the policy announcement date.
  Values: Date or blank

# University Spring Break

- *Start Date.* The date specified in the university’s academic calendar as the first day of spring break. If the university operates on a quarter schedule, spring break was recorded as the break between the winter and spring quarters. Dates were recorded regardless of the day of the week.
  Values: Date or blank
- *End Date.* The date specified in the university’s academic calendar as the last day of spring break. Similarly, if the university operates on a quarter schedule, the last day of the transition break from winter to spring quarter was used as the end date for spring break. Dates were recorded regardless of the day of the week and then standardized during data cleaning. If extended spring break dates were updated on the academic calendar and the original dates could not be located on the university website, the last day of the extended spring break was used and a note was made that the date provided correlated with a spring break extension.
  Values: Date or blank
