# Supplementary material for: COVID-19 observations and accompanying dataset of non-pharmaceutical interventions across U.S. universities, March 2020
Source: PLoS One. 2020 Oct 16;15(10):e0240786. doi: 10.1371/journal.pone.0240786 (PMC7567344; doi:10.1371/journal.pone.0240786)
Supplement: S1 File — (DOCX) [file pone.0240786.s008.docx]

| **Data Field** | **Description** |
| --- | --- |
| **Introduction**: The Integrated Postsecondary Education Data System (IPEDS), conducted by the NCES, began in 1986 and involves annual institution-level data collections. All postsecondary institutions that have a Program Participation Agreement with the Office of Postsecondary Education (OPE), U.S. Department of Education (throughout IPEDS referred to as 'Title IV') are required to report data using a web-based data collection system. IPEDS currently consists of the following components: Institutional Characteristics (IC); 12-month Enrollment (E12);Completions (C); Admissions (ADM); Student Financial Aid (SFA); Human Resources (HR) composed of Employees by Assigned Position, Fall Staff, and Salaries; Fall Enrollment (EF); Graduation Rates (GR); Outcome Measures (OM); Finance (F); and Academic Libraries (AL).  IPEDS data in this database is from the 2018 survey. | |
| unitid | Source: IPEDS  Unique identification number assigned to postsecondary institutions surveyed through the Integrated Postsecondary Education Data System (IPEDS). Also referred to as UNITID or IPEDS ID |
| School | Source: IPEDS  This annual component is the core of the IPEDS system and is required of all currently operating Title IV postsecondary institutions in the United States and other areas. As the control file for the entire IPEDS system, IC constitutes the sampling frame for all other NCES surveys of postsecondary institutions. It also helps determine the specific IPEDS screens that are shown to each institution. This component collects the basic institutional data that are necessary to sort and analyze not only the IC DATA, but also all other IPEDS DATAs. IC data are collected for the academic year, which generally extends from September of one calendar year to June of the following year. Specific data elements currently collected for each institution include: institution name. |
| State | Source: IPEDS  Full name (non abbreviated) of the state where the university is registered in IPEDS. E.g. “Virginia” |
| CensusRegion | Source: Census.gov  Census region using address of university stated in IPEDS. The Census Bureau delineates two sets of sub-national areas that are composed of states. This two-tiered system of areas consists of nine census divisions nested in four census regions. |
| CensusDivision | Source: Census.gov  Census divison using address of university stated in IPEDS. The Census Bureau delineates two sets of sub-national areas that are composed of states. This two-tiered system of areas consists of nine census divisions nested in four census regions. |
| HD2018.Institution's internet website address | Source: IPEDS  URL University main website address as listed in the Department of Education IPEDS database. |
| Voluntary_telecommute? | Source: Survey  Was remote work/telecommuting of staff/faculty encouraged?  **FALSE**   - If university still has most/all employees working on campus - If the notification states something along the lines of “employees should speak to their supervisors about whether it is best for them to work at home or not” - If staff are asked to use PTO or call-out in place of telework - If staff had to ask for approval of manager or jump through multiple hoops to get approved for telework   **TRUE**   - University tells staff that they are welcome to work from home / encourages staff to telework - University encourages staff to speak to their supervisors to start planning to work remotely - University first encourages staff to work from home, then later requires most staff to work from home - If staff are asked to stagger their time on campus, by splitting employees into two groups that alternate MWF on campus   **Blank**: Leave blank if no decision was made, that we are aware of. |
| Campus_Telecommute_Date | Source: Survey   - Use the date that correlates with the TRUE response to question 1 (“Voluntary_telecommute?”) - Use the date that they actually encourage/require telecommuting, not the date that they discuss that telecommuting is a possibility or that preparations being made for the possibility of teleworking in the future - This variable was left blank if the university did not make a decision |
| RW_State prev | Source: COVID tracking project  On the day the remote work decision was announced, the cumulative prevalence of COVID-19 positive cases within the state where the university address indicates it is located |
| Essential_personnel? | Source: Survey  Options: TRUE, FALSE, Blank  Indicated that the university limited campus access to essential/mandatory personnel only  Special criteria:   - This variable was left blank if the university did not make a decision on whether only essential personnel were permitted on campus   FALSE   - University does not specify that only essential personnel should be reporting to campus for work (other nonessential workers are allowed on campus) - School states that essential personnel have not been activated.   TRUE   - University specifies that only essential/mandatory personnel should be reported to campus for work - University states that most employees should work remotely except for employees whose job cannot be done remotely - Tiered system (i.e. essential, non-essential, stand-by), university goes to a tiered system of operation |
| Campus_essential_date | Source: Survey  The date (MM/DD/YYYY) that the university specified essential/mandatory personnel were the only individuals that should report to campus for work  Special criteria:   - If the university specified effective immediately, the date of the announcement was used - If the university specified an effective date, that specified date was used - If the university did not specify a start date, the date of the announcement was used |
| Es_State prev | Source: COVID tracking project  On the day the campus closure decision was announced, the cumulative prevalence of COVID-19 positive cases within the state where the university address indicates it is located |
| Full_semester_online? | Source: Survey  Options: TRUE, FALSE, Blank  Indicates that the university decided and announced that the remainder of the semester would be conducted in an online/distance learning format  Special criteria:   - If the decision to move the remainder of the semester online after the announcement that classes would transition to an online format, this variable was marked TRUE - This variable was marked FALSE if the university help classes online after the transition to an online format or if the decision to continue online classes was being made on an ongoing basis (interim decision points) - This variable was left blank if the university did not make a decision |
| Move_Online? | Source: Survey  Options: TRUE, FALSE, Blank  Indicates if the university announced that classes will be conducted in a online/distance learning format, whether for a few weeks or the rest of the semester  Special criteria:  FALSE   - Classes are not being conducted online or on other alternative modalities - If only preparation or discussion about the possibility of moving online is being communicated   TRUE   - It was announced that classes will be conducted online, whether for a few weeks or the rest of the semester - “Culminating Experience” (i.e. final presentation or paper to close-out semester) - All classes except those few approved (i.e. labs, studio classes) are moved online - Semester ends early with only online courses |
| Online_announced_date | Source: Survey  The date (MM/DD/YYYY) that the university announced classes would be moved to an online/distance learning format  Special criteria:   - Dates of announcements that the university was considering or preparing for a move to an online format were not used, only when the decision to move was made - In the situation that classes were moved online for only a few weeks before the university announced the movement to online for the rest of the semester, data collectors were asked to use the date at which the first move to the online format was made and then make a note of the date that the decision to extend online learning to the full semester was made - This variable was left blank if the university did not make a decision |
| OA_State prev | Source: COVID tracking project  On the day the moving learning online decision was announced, the cumulative prevalence of COVID-19 positive cases within the state where the university address indicates it is located? |
| Online_effective_date | Source: Survey  The date (MM/DD/YYYY) that classes transitioned to an online/distance learning format began, whether a date was specified by the university or the university stated “effective immediately”  Special criteria:   - If a date or “effective immediately” was not specified by the university, the date following the end of spring break or the weekend when classes would begin was used - This variable was left blank if the university did not make a decision |
| Class_suspended? | Source: Survey  Options: TRUE, FALSE, or Blank  Indicates if the university suspended/canceled classes that were scheduled in order to allow time to transition to online courses  Special criteria:   - The cancelation of classes includes the extension of spring break and/or cancelling in-person classes but continuing online classes - This variable is marked N (no) if the university does not cancel in-person or online classes, if a weekend was used to facilitate the transition to online courses, if spring break was not extended, or if the university left the decision to cancel classes to the professor’s discretion - This variable was left blank if the university did not make a decision - This variable was left blank if the university did not make a decision |
| Dorms_discouraged? | Source: Survey  Options: TRUE, FALSE, Blank  Indicates if the university encouraged students to leave or not come back to campus housing  Special criteria:  FALSE   - If university does not communicate that students should return home or stay on campus - If university recommends students stay on campus and not return home - If university requires students to fill out form to check-out of housing as an exception   TRUE   - If university requests students not return to campus, if on break - If university requests students return to permanent residency, if not on break - If university offers financial refunds of housing costs - If university requests students stay at permanent residency, if on break, but provides a time slot for students to gather their things from on-campus residency to gather personal belongings - If university states that students are welcome to return to their permanent residence or something along the lines of “we understand that most students want to go back to their permanent residences and we support this”   This variable was left blank if the university did not make a decision |
| dorms_announced_date | Source: Survey  The date (MM/DD/YYYY) that the university made an announcement encouraging students to either move out of on-campus housing and return to their permanent residence or encouraged students to remain at their permanent residence and not return to on-campus housing  This variable was left blank if the university did not make a decision |
| HA_State prev | Source: COVID tracking project  On the day the campus housing decision was announced, the cumulative prevalence of COVID-19 positive cases within the state where the university address indicates it is located |
| Study_abroad_canceled? | Source: Survey  Options: TRUE, FALSE, Blank  FALSE   - International travel is still occurring with no cancellations - University only canceled travel to CDC level countries - If the college noted that they recommend students return, but offer their full support for those that stay. Or those that only provided guidance to students about studying abroad, but no mandate - University says they encourage students to return but support their decision to stay   TRUE   - University has suspended all university sponsored traveled to all countries outside of the United States   This variable was left blank if the university did not make a decision |
| Abroad_cancel_announced_date | Source: Survey  The date (MM/DD/YYYY) that the announcement was made by the university that university-sponsored abroad trip for students is/is going to be suspended  Special criteria:   - Only announcements that all university-sponsored travel is canceled/suspended/prohibited were used, not date of announcements canceling travel to a select country, countries or region - This variable was left blank if the university did not make a decision |
| CT_State prev | Source: COVID tracking project  On the day the travel cancellation decision was announced, the cumulative prevalence of COVID-19 positive cases within the state where the university address indicates it is located |
| Abroad_cancel_effective_date | Source: Survey  The date (MM/DD/YYYY) that the university suspended university-sponsored abroad travel for students  Special criteria:   - The effective date was used when specified by the University - If the University stated “effective immediately” the date the announcement was made was the date listed - This variable was left blank if the university did not make a decision - If the university did not specify an effective date or “effective immediately”, the data collectors were asked to use the date of the announcement |
| Springbreak.start | Source: Survey of academic calendars  The date (MM/DD/YYYY) that spring break began in accordance with the university's academic calendar, regardless of day of the week   - If the date was changed in the academic calendar to reflect an extended break, data collectors were asked to put the original end date of spring break not the extended end date |
| Springbreak.end | Source: Survey of academic calendars  The date (MM/DD/YYYY) that spring break ends in accordance with the university’s academic calendar, regardless of day of the week  Special criteria:   - If the date was changed in the academic calendar to reflect an extended break, data collectors were asked to put the original end date of spring break not the extended end date |
| SOE Date State | Source: State health departments  Date a state announced a State of Emergency related to COVID |
| HD2018.Postsecondary and Title IV institution indicator | Source: IPEDS   - Title IV postsecondary institution - Institution is not active in current universe |
| HD2018.Degree-granting status | Source: IPEDS  Indicates if university grants degrees. This variable was part of the inclusion criteria.   - Degree granting |
| HD2018.Institution size category | Source: IPEDS  This indicator is derived based on the institution's total students enrolled for credit. Universities larger than 5,000 enrollment was an inclusion criteria item.   1. 5,000-9,999 2. 10,000-19,999 3. 20,000 and above |
| HD2018.Street address or post office box | Source: IPEDS  This annual component is the core of the IPEDS system and is required of all currently operating Title IV postsecondary institutions in the United States and other areas. As the control file for the entire IPEDS system, IC constitutes the sampling frame for all other NCES surveys of postsecondary institutions. It also helps determine the specific IPEDS screens that are shown to each institution. This component collects the basic institutional data that are necessary to sort and analyze not only the IC DATA, but also all other IPEDS DATAs. IC data are collected for the academic year, which generally extends from September of one calendar year to June of the following year. Specific data elements currently collected for each institution includes the institution’s address. |
| HD2018.ZIP code | Source: IPEDS  This annual component is the core of the IPEDS system and is required of all currently operating Title IV postsecondary institutions in the United States and other areas. As the control file for the entire IPEDS system, IC constitutes the sampling frame for all other NCES surveys of postsecondary institutions. It also helps determine the specific IPEDS screens that are shown to each institution. This component collects the basic institutional data that are necessary to sort and analyze not only the IC DATA, but also all other IPEDS DATAs. IC data are collected for the academic year, which generally extends from September of one calendar year to June of the following year. Specific data elements currently collected for each institution includes the institution’s zip code. |
| HD2018.Multi-institution or multi-campus organization | Source: IPEDS  University is a member of a system of coordinated institutions (multi-campus system)   1. Institution is NOT part of a multi-institution or multi-campus organization 2. Institution is part of a multi-institution or multi-campus organization |
| HD2018.Identification number of multi-institution or multi-campus organization | Source: IPEDS  Identification number of a system of coordinated institutions (multi-campus system).  Data is name of coordinated institutions, prefixed by 2 letter state abbreviation |
| HD2018.Institution grants a medical degree | Source: IPEDS  An institutional classification developed by the Andrew W. Carnegie Foundation for the Advancement of Teaching. Medical Schools and Medical Centers award most of their professional degrees in medicine. In some instances, they include other health professions programs , such as dentistry, pharmacy, or nursing. |
| HD2018.Institution has hospital | Source: IPEDS  Hospitals and/or medical centers operated by an entity for which the primary function s other than higher education |
| HD2018.State and 114TH Congressional District ID | Source: IPEDS  The Congressional house district where the address of the university in IPEDS indicates it is located. |
| House party affiliation | Source: Congress.gov  Political party affiliation of the Congressional House district for data element HD2018.State and 114TH Congressional District ID. Variable encoding:   - Empty 0 - Democrat 1 - Independent 2 - Republican 3 |
| Governor Party Affiliation | Source: National Governors Association  Political party affiliation of the governor of the state reflected in the university address. Variable encoding:   - Empty 0 - Democrat 1 - Independent 2 - Republican 3 |
| HD2018.Longitude location of institution | Source: IPEDS  Longitude and latitude values (often referred to as XY coordinates) are geographic coordinates that are used to identify the estimated location of a school campus. Longitude is the east or west angular distance from the prime meridian, with positive values going east and negative values going west. When combined with latitude, it reflects an estimation of where the school is located. Coordinate degrees, minutes, and seconds have been converted to six-digit decimal degrees. |
| HD2018.Latitude location of institution | Source: IPEDS  Longitude and latitude values (often referred to as XY coordinates) are geographic coordinates that are used to identify the estimated location of a school campus. Latitude is the north or south angular distance from the equator, with positive values going north and negative values going south. When combined with longitude, it reflects an estimation of where the school is located. Coordinate degrees, minutes, and seconds have been converted to six-digit decimal degrees. |
| IC2018.Institutional control or affiliation | Source: IPEDS  A classification of whether an institution is operated by publicly elected or appointed officials (public control) or by privately elected or appointed officials and derives its major source of funds from private sources (private control).   1. Public 2. Private not-for-profit (religious affiliation) 3. Private not-for-profit (no religious affiliation) |
| IC2018.Religious affiliation | Source: IPEDS  A classification that indicates whether a private not-for-profit institution is associated with a religious group or denomination. Private not-for-profit institutions may be either independent or religiously affiliated.   1. Baptist 2. Brethren Church 3. Christian Church (Disciples of Christ) 4. Church of God 5. Churches of Christ 6. Cumberland Presbyterian 7. Evangelical Christian 8. Interdenominational 9. Latter Day Saints (Mormon Church) 10. Lutheran Church - Missouri Synod 11. Not applicable 12. Roman Catholic 13. Southern Baptist 14. Undenominational 15. United Methodist 16. Wesleyan |
| IC2018.Study abroad | Source: IPEDS  Arrangement by which a student completes part of the college program studying in another country. Can be at a campus abroad or through a cooperative agreement with some other U.S. college or an institution of another country. |
| DRVEF2018.Percent of first-time undergraduates - foreign countries | Source: IPEDS  This annual component of IPEDS collects data on the number of students enrolled in the fall at postsecondary institutions. Students reported are those enrolled in courses creditable toward a degree or other recognized postsecondary credential; students enrolled in courses that are part of a vocational or occupational program, including those enrolled in off-campus or extension centers; and high school students taking regular college courses for credit. Institutions report annually the number of full- and part-time students, by gender, race/ethnicity, and level (undergraduate, graduate, first-professional); the total number of undergraduate entering students (first-time, full-and part-time students, transfer-ins, and non-degree students); and retention rates. In even-numbered years, **data are collected for state of residence of first-time students and for the number of those students who graduated from high school or received high school equivalent certificates in the past 12 months.** Also in even-numbered years, 4-year institutions are required to provide enrollment data by gender, race/ethnicity, and level for selected fields of study. In odd-numbered years, data are collected for enrollment by age category by student level and gender. |
| HD2018.Degree of urbanization (Urban-centric locale) | Source: IPEDS  A code representing the urbanicity (city/suburb/rural) by population size of the institution's location. This urbancentric locale code was assigned through a methodology developed by the U.S. Census Bureau's Population Division in 2005. The urban-centric locale codes apply current geographic concepts to the original NCES locale codes used on IPEDS files through 2004.   1. City: Large 2. City: Midsize 3. City: Small 4. Rural: Fringe 5. Rural: Remote 6. Suburb: Large 7. Suburb: Midsize 8. Suburb: Small 9. Town: Distant 10. Town: Fringe 11. Town: Remote |
| HD2018.Carnegie Classification 2018: Graduate Instructional Program | Source: IPEDS  As a companion to the Undergraduate Instructional Program classification, this classification examines the nature of graduate education, with a special focus on the mix of graduate programs. In this classification, a single graduate-level degree qualifies an institution for inclusion.The classification is based on the level of graduate degrees awarded master's/professional or doctoral), the number of fields represented by the degrees awarded, and the mix or concentration of degrees by broad disciplinary domain. The classification has two parts: one for institutions that do not award the doctorate, and one for doctoral-level institutions (based on the record of degree conferrals, not program offerings). Within each group, institutions are then classified with respect to the breadth of graduate offerings and the concentration of degrees in certain fields or combinations of fields. For a complete description and technical details visit the Carnegie Foundation Website at http://www.carnegiefoundation.org/classifications   1. Not classified (Exclusively Undergraduate) 2. Postbaccalaureate: Arts & sciences-dominant 3. Postbaccalaureate: Business-dominant, with Arts & Sciences 4. Postbaccalaureate: Business-dominant, with other professional programs 5. Postbaccalaureate: Comprehensive programs 6. Postbaccalaureate: Education-dominant, with Arts & Sciences 7. Postbaccalaureate: Education-dominant, with other professional programs 8. Postbaccalaureate: Other-dominant, with Arts & Sciences 9. Postbaccalaureate: Other-dominant, with other professional programs 10. Postbaccalaureate: Single program-Other 11. Research Doctoral: Comprehensive programs, no medical/veterinary school 12. Research Doctoral: Comprehensive programs, with medical/veterinary school 13. Research Doctoral: Professional-dominant 14. Research Doctoral: Single program-Education 15. Research Doctoral: Single program-Other 16. Research Doctoral: STEM-dominant 17. #N/A |
| HD2018.Carnegie Classification 2018: Size and Setting | Source: IPEDS  This classification describes institutions' size and residential character. Because residential character applies to the undergraduate student body, exclusively graduate/professional institutions are not included. For a complete description and technical details visit the Carnegie Foundation Website at <http://www.carnegiefoundation.org/classifications>   1. Four-year, large, highly residential 2. Four-year, large, primarily nonresidential 3. Four-year, large, primarily residential 4. Four-year, medium, highly residential 5. Four-year, medium, primarily nonresidential 6. Four-year, medium, primarily residential 7. Four-year, small, highly residential 8. Four-year, small, primarily nonresidential 9. Not applicable, not in Carnegie universe (not accredited or nondegree-granting) |
| DRVEF2018.Full-time enrollment | Source: IPEDS  The number of FTE students is calculated based on fall student headcounts as reported by the institution on the IPEDS Enrollment (EF) component (Part A). The full-time equivalent (headcount) of the institution's part-time enrollment is estimated by multiplying the factors noted below times the part-time headcount. These are then added to the full-time enrollment headcounts to obtain an FTE for all students enrolled in the fall. |
| DRVEF2018.Percent of total enrollment that are American Indian or Alaska Native | Source: IPEDS  A person having origins in any of the original peoples of North and South America (including Central America) who maintains cultural identification through tribal affiliation or community attachment. |
| DRVEF2018.Percent of total enrollment that are Asian | Source: IPEDS  A person having origins in any of the original peoples of the Far East, Southeast Asia, or the Indian Subcontinent, including, for example, Cambodia, China, India, Japan, Korea, Malaysia, Pakistan, the Philippine Islands, Thailand, and Vietnam. |
| DRVEF2018.Percent of total enrollment that are Black or African American | Source: IPEDS  Percent of students having origins in any of the black racial groups of Africa. |
| DRVEF2018.Percent of total enrollment that are Hispanic/Latino | Source: IPEDS  Percent of students of Cuban, Mexican, Puerto Rican, South or Central American, or other Spanish culture or origin, regardless of race. |
| DRVEF2018.Percent of total enrollment that are Native Hawaiian or Other Pacific Islander | Source: IPEDS  Percent of students having origins in any of the original peoples of Hawaii, Guam, Samoa, or other Pacific Islands. |
| DRVEF2018.Percent of total enrollment that are White | Source: IPEDS  Percent of students having origins in any of the original peoples of Europe, the Middle East, or North Africa. |
| DRVEF2018.Percent of total enrollment that are two or more races | Source: IPEDS  Percent of students of two or more races. These data were collected in the Enrollment component prior to the 2007 IPEDS collection. Data are collected for the entire 12-month academic year, while enrollment data collected in the Fall Enrollment component are fall data. Institutions report an unduplicated head count for the total number of students by **gender,** attendance status (full-time, part-time), race/ethnicity, and level (undergraduate, graduate, first-professional) enrolled throughout the reporting period. Students included are those enrolled in any courses leading to a degree or other recognized postsecondary credential, as well as those enrolled in courses that are part of a terminal vocational or occupational program. Institutions also report the total instructional activity for the same 12-month period for both undergraduate and graduate programs. Instructional activity data are reported in units of clock hours or credit hours. |
| DRVEF2018.Percent of total enrollment that are Race/ethnicity unknown | Source: IPEDS  Percent of students whose race and ethnicity are not known. |
| DRVEF2018.Percent of total enrollment that are Nonresident Alien | Source: IPEDS  Percent of students who are not a citizen or national of the United States and who is in this country on a visa or temporary basis and does not have the right to remain indefinitely. |
| DRVEF2018.Percent of total enrollment that are women | Source: IPEDS  Percent of students who are female. These data were collected in the Enrollment component prior to the 2007 IPEDS collection. Data are collected for the entire 12-month academic year, while enrollment data collected in the Fall Enrollment component are fall data. Institutions report an unduplicated head count for the total number of students by **gender,** attendance status (full-time, part-time), race/ethnicity, and level (undergraduate, graduate, first-professional) enrolled throughout the reporting period. Students included are those enrolled in any courses leading to a degree or other recognized postsecondary credential, as well as those enrolled in courses that are part of a terminal vocational or occupational program. Institutions also report the total instructional activity for the same 12-month period for both undergraduate and graduate programs. Instructional activity data are reported in units of clock hours or credit hours. |
| S2018_OC.Grand total | Source: IPEDS  Count of university all faculty and staff. These data were collected in the Enrollment component prior to the 2007 IPEDS collection. Data are collected for the entire 12-month academic year, while enrollment data collected in the Fall Enrollment component are fall data. Institutions report an unduplicated head count for the total **number of students** by gender, attendance status (full-time, part-time), race/ethnicity, and level (undergraduate, graduate, first-professional) enrolled throughout the reporting period. Students included are those enrolled in any courses leading to a degree or other recognized postsecondary credential, as well as those enrolled in courses that are part of a terminal vocational or occupational program. Institutions also report the total instructional activity for the same 12-month period for both undergraduate and graduate programs. Instructional activity data are reported in units of clock hours or credit hours. |
| S2018_OC.Grand total men | Source: IPEDS  Count of faculty and staff that are men. These data were collected in the Enrollment component prior to the 2007 IPEDS collection. Data are collected for the entire 12-month academic year, while enrollment data collected in the Fall Enrollment component are fall data. Institutions report an unduplicated head count for the total number of students by **gender**, attendance status (full-time, part-time), race/ethnicity, and level (undergraduate, graduate, first-professional) enrolled throughout the reporting period. Students included are those enrolled in any courses leading to a degree or other recognized postsecondary credential, as well as those enrolled in courses that are part of a terminal vocational or occupational program. Institutions also report the total instructional activity for the same 12-month period for both undergraduate and graduate programs. Instructional activity data are reported in units of clock hours or credit hours. |
| S2018_OC.Grand total women | Source: IPEDS  Count of faculty and staff that are women. These data were collected in the Enrollment component prior to the 2007 IPEDS collection. Data are collected for the entire 12-month academic year, while enrollment data collected in the Fall Enrollment component are fall data. Institutions report an unduplicated head count for the total number of students by **gender**, attendance status (full-time, part-time), race/ethnicity, and level (undergraduate, graduate, first-professional) enrolled throughout the reporting period. Students included are those enrolled in any courses leading to a degree or other recognized postsecondary credential, as well as those enrolled in courses that are part of a terminal vocational or occupational program. Institutions also report the total instructional activity for the same 12-month period for both undergraduate and graduate programs. Instructional activity data are reported in units of clock hours or credit hours. |
| S2018_OC.American Indian or Alaska Native total | Source: IPEDS  Count of faculty and staff that have origins in any of the original peoples of North and South America (including Central America) who maintains cultural identification through tribal affiliation or community attachment. |
| S2018_OC.Asian total | Source: IPEDS  Count of faculty and staff that have origins in any of the original peoples of the Far East, Southeast Asia, or the Indian Subcontinent, including, for example, Cambodia, China, India, Japan, Korea, Malaysia, Pakistan, the Philippine Islands, Thailand, and Vietnam. |
| S2018_OC.Black or African American total | Source: IPEDS  Count of faculty and staff that have origins in any of the black racial groups of Africa. |
| S2018_OC.Hispanic or Latino total | Source: IPEDS  Count of faculty and staff that have Cuban, Mexican, Puerto Rican, South or Central American, or other Spanish culture or origin, regardless of race. |
| S2018_OC.Native Hawaiian or Other Pacific Islander total | Source: IPEDS  Count of faculty and staff that have origins in any of the original peoples of Hawaii, Guam, Samoa, or other Pacific Islands. |
| S2018_OC.White total | Source: IPEDS  Count of faculty and staff that have origins in any of the original peoples of Europe, the Middle East, or North Africa. |
| S2018_OC.Two or more races total | Source: IPEDS  Count of faculty and staff of 2 or more races. These data were collected in the Enrollment component prior to the 2007 IPEDS collection. Data are collected for the entire 12-month academic year, while enrollment data collected in the Fall Enrollment component are fall data. Institutions report an unduplicated head count for the total number of students by **gender,** attendance status (full-time, part-time), race/ethnicity, and level (undergraduate, graduate, first-professional) enrolled throughout the reporting period. Students included are those enrolled in any courses leading to a degree or other recognized postsecondary credential, as well as those enrolled in courses that are part of a terminal vocational or occupational program. Institutions also report the total instructional activity for the same 12-month period for both undergraduate and graduate programs. Instructional activity data are reported in units of clock hours or credit hours. |
| S2018_OC.Race/ethnicity unknown total | Source: IPEDS  The count of staff whose race and ethnicity are not known. |
| S2018_OC.Nonresident alien total | Source: IPEDS  The count of faculty and staff that are not a citizen or national of the United States and who is in this country on a visa or temporary basis and do not have the right to remain indefinitely. |
